# A comparison of contrast transthoracic echocardiography and contrast transcranial Doppler in cryptogenic stroke patients with patent foramen ovale

**DOI:** 10.1002/brb3.1283

**Published:** 2019-04-02

**Authors:** Jie Chen, Luyun Chen, Wangwang Hu, Xianda Ni, Zengrui Zhang, Xiaowen Feng, Zijian Fan, Cuiping Chen, Fengzhen Qiu, Bei Shao

**Affiliations:** ^1^ Department of Neurology First Affiliated Hospital of Wenzhou Medical University Wenzhou China; ^2^ Department of Ultrasonography First Affiliated Hospital of Wenzhou Medical University Wenzhou China

**Keywords:** contrast transcranial Doppler, contrast transthoracic echocardiography, cryptogenic stroke, patent foramen ovale, transesophageal echocardiography

## Abstract

**Objective:**

In recent years, increasing attention has been paid to cryptogenic stroke (CS) caused by the patent foramen ovale (PFO). This study aims to compare contrast transthoracic echocardiography (cTTE) and contrast transcranial Doppler (cTCD) to determine whether cTTE is more suitable and reliable than cTCD for clinical use.

**Methods:**

From March 2017 to May 2018, patients who suffered from migraines, stroke, hypomnesis, or asymptomatic stroke found casually were included in our study. Patients with CS were semirandomly divided into two groups (cTTE and cTCD) according to the date of the outpatient visit. Patients with either of the examination above found positive were selected to finish transesophageal echocardiography (TEE).

**Results:**

In our study, the sensitivities of group cTTE positive (group cTTE+) and group cTCD positive (group cTCD+) did not have any statistical difference (89% vs. 80%, *p* = 0.236). Focusing on group cTCD+, we discovered that the semiquantitative shunt grading was not correlated with whether a PFO was present or not (*p* = 0.194). However, once the PFO has been diagnosed, the shunt grading was shown to be related to the width of the gaps (*p* = 0.032, *p*_deviation_ = 0.03).

**Conclusion:**

Both cTTE and the cTCD can be used for preliminary PFO findings. The semiquantitative shunt grading of cTCD and cTTE can suggest the size of the PFO and the next course of treatment. The cTTE may be more significant to a safe PFO (a PFO does not have right‐to‐left shunts, RLSs). Combining cTTE and TEE could help diagnose PFO and assess CS risk.

## 1 INTRODUCTION

The foramen ovale is a physiological channel of the atrial septum. At birth, with the development of the lungs, the foramen ovale is functionally closed (Handke, Harloff, Bode, & Geibel, 2009). Patent foramen ovale (PFO) arises when children older than 3 years do not have their foramen ovale closed. Approximately 10%–35% of adults have a PFO in the general population, but the frequency is even higher in cryptogenic stroke (CS) patients (Anzola, Giusti Del Giardino, & Piras, 2010; Belvis et al., 2006; Katsanos et al., 2014; Souteyrand et al., 2006; Yue, Zhai, & Wei, 2014). Since the PFO shunt is too small, PFO had long been considered a nonserious clinical presentation (Hara et al., 2005; Zhao, Cheng, Zhang, Li, & Wang, 2017). In recent years, however, increasing studies have shown that PFO patients have a higher morbidity of stroke, migraines, and other relevant diseases than normal population. Hence, more attention has been paid in particular to the relationship between PFO and CS (Anzola et al., 2010; Handke et al., 2009; Thaler & Wahl, 2012).

Three theories have been proposed to explain the role of the PFO in CS. The first is called the paradoxical embolism theory, in which an embolus from a deep venous thrombosis goes through the PFO and enters the arterial system. The second is the arrhythmogenic theory, where the PFO induces arrhythmias by an atrial vulnerability mechanism (Karine et al., 2000). Finally, the thrombogenic theory postulates that thrombi are generated in situ (Belvis et al., 2006). With the introduction of TTE and transesophageal echocardiography (TEE) in the 1980s and 1990s, PFO was not difficult to diagnose (Lynch, Schuchard, Gross, & Wann, 1984). At present, the most common procedures for detecting a PFO are contrast transcranial Doppler (cTCD), contrast transthoracic echocardiography (cTTE), and TEE (Handke et al., 2009; Soliman et al., 2007; Yue et al., 2014; Zhao et al., 2017). The cTCD has the highest sensitivity among the three. It is the cheapest but does not provide any information about the anatomy of the atrial septum and associated structures (Devuyst, Despland, Bogousslavsky, & Jeanrenaud, 1997; Handke et al., 2009; Yue et al., 2014). The cTTE is more superior and intuitive. Yet, there is no uniform definition regarding the number of microbubbles (MBs) thus it is unable to give the size of a PFO (Hausmann, Mügge, & Daniel, 1995; Homma et al., 1994; Martín et al., 2002). Transesophageal echocardiography is regarded as a gold standard. (Pearson, Labovitz, Tatineni, & Gomez, 1991; Schneider et al., 1996). However, it has the disadvantage of being semi‐invasive and may be difficult for stroke patients with swallowing difficulty and/or poor cooperation (Belvis et al., 2006; Jong‐Won et al., 2001). Therefore, this study aims to compare cTTE and cTCD to determine whether cTTE is more suitable and reliable than cTCD for clinical use.

## 2 METHODS

### 2.1 Study population

From March 2017 to May 2018, patients who suffered migraines, stroke, hypomnesis, or asymptomatic stroke found by a brain magnetic resonance imaging (MRI) were consecutively recruited into this prospective research study.

A previous study had highlighted the relationship between migraine and CS (West et al., 2018). Hypomnesis, similar to vascular dementia (VD), is defined as some patients who had a poorer memory than before, in which their daily life or work was affected. Demographic information (age, gender) and medical history (hypertension, hyperlipidemia, and diabetes) were collected for all patients. Laboratory tests of all patients such as blood routine, biochemistry, and homocysteine were tested in the hospital's biochemistry department. All patients were required to finish correlative imaging examination, such as carotid ultrasound, transcranial Doppler (TCD), MRI, electrocardiography (ECG), and echocardiography. By analyzing MRI, especially T2‐weighted MRI (T2WI) and fluid‐attenuated inversion recovery (FLAIR), we found a subset of patients who had no sufficient evidence to support atherosclerotic and cardiogenic events, while MRI showed more subcortical frontal and parietal small lesions (Huang, Shao, Ni, & Li, 2014; Kim et al., 2013) which are suspected of PFO.

Our exclusion criteria were as follows: (a) Patients who had cardiac disease such as atrial fibrillation or valvular heart disease found either in the past or present; (b) Carotid atherosclerosis patients whose plaque location was consistent with MRI lesions (Dieleman et al., 2016; Gao, Yu, & Liu, 2014); (c) Patients who had high risk factors for small atherosclerosis such as hypertension or diabetes with deep perforators lesions on MRI; (d) MRI with no lesions. Due to the large number of outpatients and dozens of doctors providing treatment every half day, it is difficult to have centralized management. Thus, we decided to group the patients semirandomly according to the distribution of the total number of outpatients. The cTTE were provided on Monday and Wednesday, while cTCD were offered on other workdays. A total of 361 patients were enrolled in the study. Ninety‐seven subjects who were cTTE+ or cTCD+ were selected to have a TEE performed. Hypertension was defined as high blood pressure (systolic blood pressure greater than or equal to 140 mm Hg or diastolic blood pressure greater than or equal to 90 mm Hg) or the taking of antihypertensive agents. Diabetes was defined as a high fasting blood glucose (FBG) level (higher than or equal to 7.0 mmol/L) or the taking of hypoglycemic agents. Hyperlipidemia was defined as a high level of serum total cholesterol (>5.6 mmol/L), triglycerides (>1.7 mmol/L), low‐density lipoprotein cholesterol (>3.4 mmol/L), high‐density lipoprotein (<0.9 mmol/L), or treatment with antihyperlipidemic agents after diagnosis of hyperlipidemia (Chen et al., 2018).

From the TEE results, we calculated the sensitivities of group cTCD+ and group cTTE+. Then in group cTCD+, the patients were divided into two groups: TEE positive (TEE+) and TEE negative (TEE−). A comparison of the semiquantitative shunt grading between the two groups was performed. Moreover, in patients who were cTCD+ and TEE+, the relationship between the semiquantitative shunt grading and the gap of PFO was performed.

### 2.2 Imaging

#### 2.2.1 Transesophageal echocardiography

Transesophageal echocardiography was performed with a Philips iE Elite Ultrasound machine using a X7‐2t multiplane transesophageal probe. All patients were fasted and local pharyngeal anesthesia was achieved with the administration of adequate amounts of 0.02% oral lidocaine before the TEE examination. The area of the atrial septum where the foramen ovale located was analyzed from various angles to find the appearance of the left‐to‐right shunts (LRSs). The width of gap between the atrial septum and PFO valve was recorded.

#### 2.2.2 Contrast transthoracic echocardiography

The cTTE was performed with the Philips iE Elite Ultrasound machine using a X5‐1 probe. Two 10‐ml syringes were prepared in advance for the purpose of obtaining activated saline (a mixture of 9 ml saline and 1 ml air). All patients were required to perform the Valsalva maneuver (VM) before the beginning of the test. If effective, the atrial septal protrusion can be observed in the left atrium after exhalation. Since the appearance of MB by ultrasound is intuitive and clear, a PFO was diagnosed once any MB was discovered in the left atrium within three cycles after the contrast had appeared in the right atrium.

#### 2.2.3 Contrast transcranial Doppler

The cTCD was performed with an Elica TCD machine using a 1.6 MHz probe. The left middle cerebral artery (LMCA) was monitored through the temporal bone window. Similar to cTTE, activated saline and VM were needed for this test. The VM was considered effective when there is a 25% decrease of MCA flow velocity (Zetola et al.., 2012). The modified quantification criteria used were based on the Consensus Conference of Venice (Serena et al., 1998). The test was seen as positive if at least one “hit” was recorded within 10 s after the injection. The results were classified as follows: 0 hit, negative (0); 1–10 hits, small shunt (I); 10–25 hits, medium shunt (II); and > 25 hits including “curtain” effect (the hits were too many to calculate), large shunt (III); (González‐Alujas et al., 2011; Handke et al., 2009; He et al., 2017; Souteyrand et al., 2006).

In this prospective study, only anonymized data previously acquired, as part of the patient workup or for service evaluation purposes, were used. The study was approved by the ethics committee of the First Affiliated Hospital of Wenzhou Medical University. All patients or their legal representations sign the informed consent before inclusion in this study.

### 2.3 Statistics

Continuous variables were expressed as the mean value ± standard deviation or medians with interquartile ranges according to the normality of data distribution. Categorical variables were expressed as counts and proportions. The sensitivities of the groups and the comparison of shunt grading between the two groups were analyzed using the Pearson chi‐squared test. The relationship between the shunt grading and the gap was analyzed using the linear‐by‐linear association. Statistical significance was set at a *p* value <0.05.

All statistical analyses were performed using spss version 24.0 (SPSS Inc, Chicago, IL).

## 3 RESULTS

**Figure 1:**
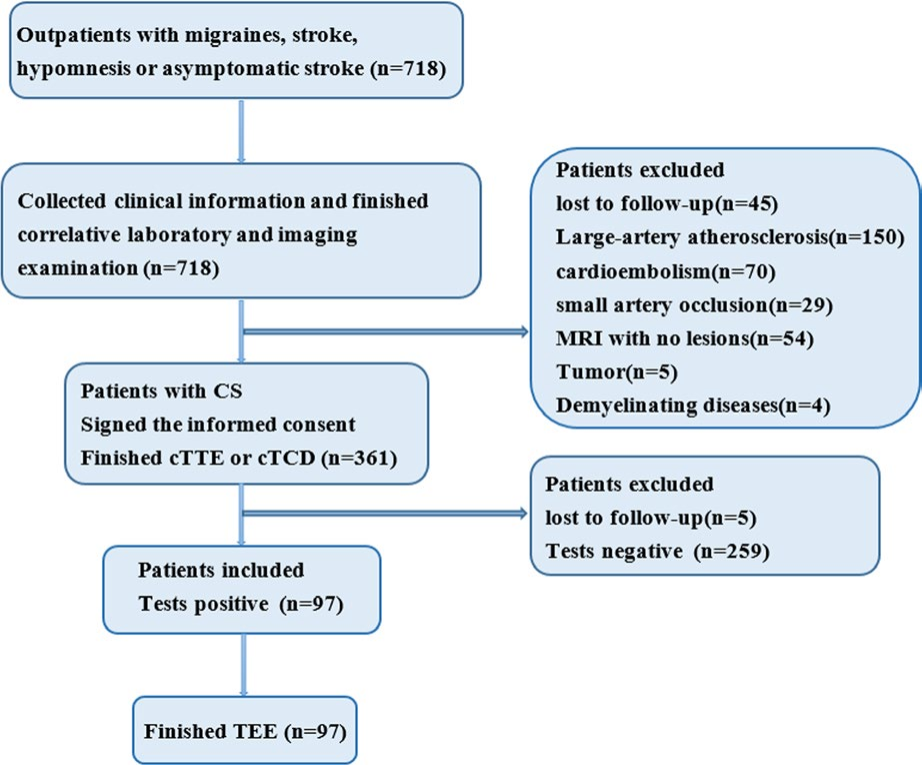
Flow diagram

We recruited a total of 718 patients. After screening through clinical information, laboratory and imaging examination, 361 patients were eligible for our study (Figure 1). Of these 361 patients, 60/205 patients were cTCD+, 37/151 were cTTE+, and five were lost to follow‐up. Baseline characteristics of 356 patients are shown in Table 1. A total of 97 patients testing positive for cTCD and cTTE consist of 41 men and 56 women (median age 48, interquartile range 42–56). Among those, in terms of initial symptoms, 46 suffered from migraines or dizziness, 25 had stroke, 17 had asymptomatic stroke found by a brain MRI, four hypomnesis, three syncope, one limb shaking, and one abdominal discomfort without digestive diseases.

**Table 1:** Baseline patient characteristics

Characteristics	*n* = 356
Age (years; median, IQR)	44 (40–55)
Male (%)	147 (41.3)
Hypertension (%)	106 (29.8)
Hyperlipidemia (%)	40 (11.2)
Diabetes (%)	103 (28.9)
Carotid ultrasound (%)	
Normal	250 (70.1)
Thickened	55 (15.5)
Carotid plaque	51 (14.4)
RBC (median, IQR)	4.37 (4.13–4.70)
WBC (median, IQR)	5.75 (4.72–6.98)
PLT (median, IQR)	210.00 (182.75–266.25)
HB (median, IQR)	130.00 (121.00–142.25)
TC (median, IQR)	4.36 (3.67–4.92)
TG (median, IQR)	1.24 (0.84–1.61)
HDL (median, IQR)	1.16 (0.93–1.46)
LDL (median, IQR)	2.50 (1.97–2.88)
FBG (median, IQR)	5.20 (4.55–5.60)
Hcy (median, IQR)	11.00 (9.00–12.50)

Note

FBG: fasting blood sugar; HB: hemoglobin; Hcy: homocysteine; HDL: high‐density lipoprotein; IQR: interquartile range; LDL: low‐density lipoprotein; PLT: platelet; RBC: red blood cell; TC: total cholesterol; TG: triglyceride; WBC: white blood cell.

Using the TEE as a gold standard, the sensitivities of the cTTE+ and cTCD+ groups did not have statistical difference (89% vs. 80%, *p* = 0.236) (Table 2). Of the 97 patients, 19 of them required initiatively to finish both cTTE and cTCD after they were informed our trial. Then they were semirandomly divided into two groups according to our date grouping criteria. So there were 19 patients who finished all three examinations, 11/19 were both cTCD and cTTE positive (Table 3). Surprisingly, two of the 11 patients who were tested cTCD and cTTE positive had been found TEE negative.

**Table 2:** Comparison of sensitivities for two groups

	TEE+	TEE−	Total
cTCD+	48	12	60
cTTE+	33	4	37
Total	81	16	97

Note

cTCD+: contrast transcranial Doppler positive; cTTE+: contrast transthoracic echocardiography positive; TEE+: transesophageal echocardiography positive; cTTE−: contrast Transthoracic echocardiography negative.

*p* = 0.236.

**Table 3:** Comparison of tests in 19 patients

	cTTE+	cTTE−	Total
cTCD+	11	1	12
cTCD−	7	0	7
Total	18	1	19

Note

cTCD+: contrast transcranial Doppler positive; cTTE+: contrast transthoracic echocardiography positive; cTCD−: contrast transcranial Doppler negative; cTTE−: contrast transthoracic echocardiography negative.

**Figure 2:**
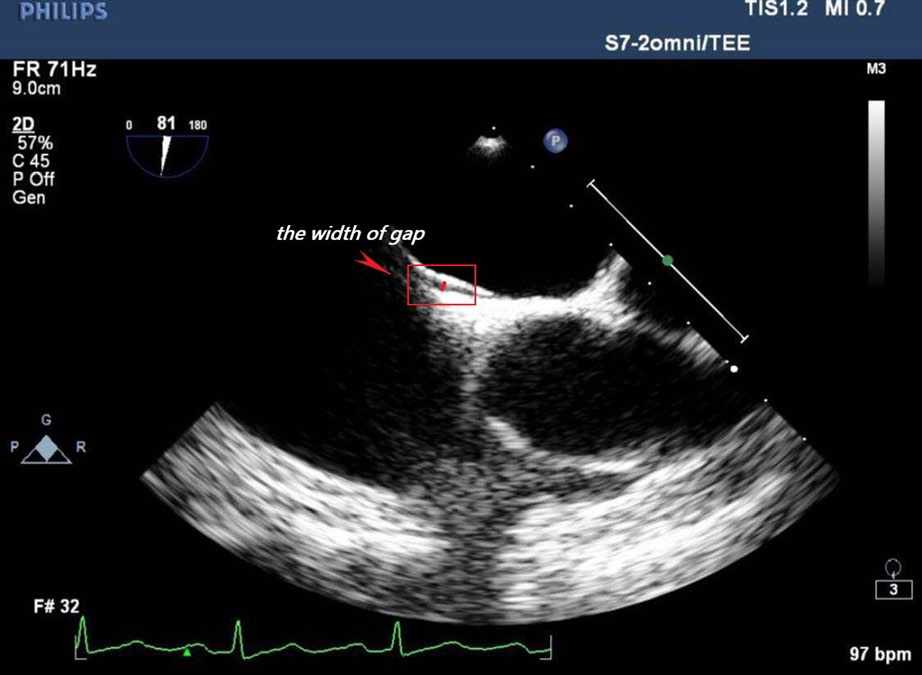
The width of gap

Next, focusing on group cTCD+ then using TEE results for grouping, we compared the semiquantitative shunt grading between the two groups and found no statistical difference(*p* = 0.194) (Table 4). For patients who were TEE+, we further grouped them by the width of their PFO gaps (Figure 2) and found the semiquantitative shunt grading to be related to the gaps but this was not a straight line (*p* = 0.032, *p*_deviation_ = 0.03) (Table 5). In short, we discovered that the semiquantitative shunt grading had no relation to whether a PFO was present or not. However, once the PFO has been diagnosed, the shunt grading related to the gaps and the specific quantitative relationship became unclear.

**Table 4:** A comparison of the semiquantitative shunt grading between the two groups

	I	II	III	Total
TEE+	27	11	10	48
TEE−	10	1	1	12
Total	37	12	11	60

Note

TEE+: transesophageal echocardiography positive; TEE−: transesophageal echocardiography negative; I: 1–10 hits small shunt; II: 10–25 hits medium shunt; III: >25 hits including “curtain” effect.

*p* = 0.194.

**Table 5:** Relationship between the semiquantitative shunt grading and the width of the gap

	*d* ≤ 1 mm	1 mm < *d *≤ 2 mm	*d* > 2 mm	Total
I	10	16	1	27
II	1	7	3	11
III	3	7	0	10
Total	14	30	4	48

Note

I: 1–10 hits small shunt; II: 10–25 hits medium shunt; III: >25 hits including “curtain” effect; *d*: the width of gap between the valve of foramen ovale and the atrial septum (unit: mm).

*p* = 0.032, *p*_deviation_ = 0.03.

## 4 DISCUSSION

From our results, cTCD and cTTE have similar sensitivities and could be used to filter preliminarily PFO cases. Maffè et al had reported that cTTE and cTCD had comparable sensitivity compared with TEE, with TEE as the reference standard, 89% for TTE, and 85% for TCD (Kühl et al., 1999; Maffè et al., 2010; Souteyrand et al., 2006). These are consistent with the results of our present study (González‐Alujas et al., 2011; Jong‐Won et al., 2001; Zhao et al., 2015).

**Figure 3:**
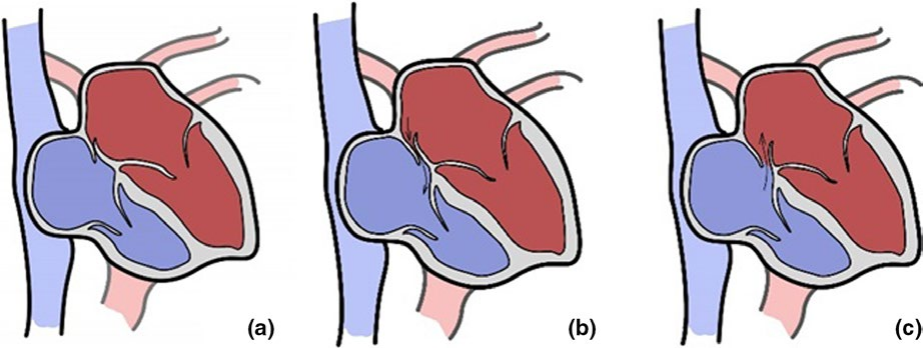
(a) Most patent foramen ovale (PFO) was a physiological closure at rest without shunts found by transesophageal echocardiography (TEE). (b) A few PFO was incomplete closure at rest and could be found left‐to‐right shunts by TEE. (c), By doing a Valsalva maneuver, the development of a right‐to‐left atrial pressure gradient results in right‐to‐left shunts, and the increased pressure of right atrial could make the PFO width lager

The hemodynamic characteristics of PFO were not mentioned in most studies. Transesophageal echocardiography could observe the anatomy of the atrial septum and associated structures. Normally, the left atrium has a higher pressure than the right atrium. In most PFO patients, we could only see the gap but no shunts by TEE because of its physiological closure (Figure 3a). In a few PFO patients, the appearance of the LRSs of the atrial level of diastole could be found by TEE through the incomplete closure of the PFO (Figure 3b). When coughing or doing a sustained VM (Jauss, Kaps, Keberle, Haberbosch, & Dorndorf, 1994; Soliman et al., 2007), the pressure of the right atrium would rise abruptly. The development of a right‐to‐left atrial pressure gradient results in right‐to‐left shunts (RLSs) (Figure 3c). Tobe et al found that RLSs during VM might be more predictive than at rest (Tobe, Bogiatzi, Munoz, Tamayo, & Spence, 2016). Usually, we do not perform VM because of poor cooperation during the TEE. Therefore, TEE is thought to be more sensitive to LRSs in rest, and does not seem to be applicable to transient RLSs if the tiny PFO could only be observed by VM (Kronik, Slany, & Moesslacher, 1979; Soliman et al., 2007). To overcome this defect, in recent years, TEE with contrast (cTEE) has been promoted for replacing TEE as the gold standard. However, Li et al suggested that cTEE, both in terms of sensitivity and revealing severity, was lower than that of cTTE (Thanigaraj, Valika, Zajarias, Lasala, & Perez, 2005; Yue et al., 2014), owing to the use of an anesthetic and then intubation during the TEE examination, which may have affected the patient's cooperation while performing the VM (González‐Alujas et al., 2011; He et al., 2017). Owing to the disadvantages above, cTEE should not be considered as the gold standard in our opinion.

Ultrasound is uniquely sensitive to MBs. In the case of a PFO, cTCD showed an injection‐detection MBs mean latency in MCA of less than 11 s (Devuyst et al., 1997), while MBs were noted in the left atrium within three to five cardiac cycles after first appearance in the right atrium for cTTE (Jauss et al., 1994; Soliman et al., 2007). Since contrast with a diameter >9 mm do not pass the pulmonary capillary circulation, any appearance of intravenously injected MBs in the time window is considered positive for an RLS. Some papers have mentioned the high false positives of cTCD. Goutman et al performed a study in 502 patients, and 63 were found to be positive cTCD with negative TTE and/or TEE. Eleven of the 63 patients were evaluated for the malignancies, a pulmonary arteriovenous malformation (PAVM), parietal AVM, arteriovenous (AV) fistula thrombus, and indeterminate reasons (Goutman, Katzan, & Gupta, 2013). Those extracardiac shunts can also cause large shunts and have been associated with a higher risk of recurrent stroke (Belvis et al., 2006; Chimowitz et al., 1991; Goutman et al., 2013; Soliman et al., 2007).

There is no uniform definition regarding the number of MBs appearance in the left atrium of cTTE in the literature，so we did not perform a semiquantitative analysis (Hausmann et al., 1995; Homma et al., 1994; Martín et al., 2002). According to the quantification criteria of the Consensus Conference of Venice (Jauss & Zanette, 2000), we conducted a semiquantitative study of cTCD. In our data, we discovered that the semiquantitative shunt grading had nothing to do with the presence of PFO. However, once the PFO has been diagnosed, the shunt grading was related to the gaps and the specific quantitative relationship became unclear. There are two possible explanations for this. One, if the number of patients was large enough, the shunt grading and the gaps might be a linear correlation. Two, the PFO gap measured by TEE was not the real width. The increased pressure of right atrium could make the PFO width lager (Figure 3c). Meanwhile, we hypothesized that if we did semiquantitative analysis between the shunt grading of cTTE and the gaps, the result should be same as cTCD. González‐Alujas et al once performed a survey where all patients with a PFO >4 mm had a moderate or severe shunt (González‐Alujas et al., 2011). Therefore, for the PFO patients, periodic follow‐up and postoperative re‐examination could be done by cTCD and cTTE instead of TEE (Di et al., 1993), which can improve patient experience and comfort.

The cTTE is limited in patients who have decreased echogenicity, sometimes VM could lead to a further decrease in image quality. For TCD, the absence of cardiac visualization and calcified temporal window prevented the use of it (Souteyrand et al., 2006). It was reported that the combination of cTCD and cTTE could greatly improve the detection rate of PFO (Souteyrand et al., 2006). We finished two tests in 19 patients out of which, two cases were both cTCD and cTTE positive but TEE negative. Possible reasons are as follows: (a) The foramen ovale was too small or always tightly closed at rest, so it could not be detected by TEE (Caputi et al., 2009; Clarke, Timperley, & Kelion, 2004; He et al., 2017); (b) The presence of pulmonary arteriovenous malformation (PAVM) or other extracardiac shunts (Droste et al., 1999; Yue et al., 2014) could require further examination to exclude other causes. The paradoxical embolism theory explains that for patients who are cTTE negative, RLSs do not occur that is, PFO that does not cause CS (“safe PFO”). But based on the other two theories, such patients can also be assessed according to risk of paradoxical embolism (RoPE) score (Thaler & Wahl, 2012), and the probability of CS with a score ≤3 (total 10) owing to PFO is 0% (Kent et al., 2013). It seems that the cTTE is more significant in discovering whether the PFO is safe or not. Thus, combining cTTE and TEE could make for more accurate diagnosing of PFO and assessing CS risk.

Our study has its limitations. Our research is a single‐center study and sample size may not be large enough to represent the general CS population. We also had not performed semiquantitative analysis of cTTE. Furthermore, the large mobility of an outpatient setting was not conducive to management and lost to follow‐up bias is difficult to control. Lastly, we did not do further tests on patients with negative result as we considered these patients to be PFO‐safe.

## 5 CONCLUSION

In summary, both cTTE and the cTCD have high sensitivities, and can be used to filter preliminarily PFO cases. The semiquantitative shunt grading of the cTCD and the cTTE could suggest the size of the PFO and could be used in the next step in treatment such as periodic follow‐up and postoperative re‐examination. To some extent, the cTTE may be more significant for determining a safe PFO. Thus, combining cTTE and TEE for clinical use could help to better diagnose PFO and assess CS risk.

## CONFLICT OF INTEREST

None declared.
